# Can oral corticosteroids reduce the severity or duration of an acute cough, and the associated National Health Service and societal costs, in adults presenting to primary care? Study protocol for a randomised controlled trial

**DOI:** 10.1186/s13063-015-0569-5

**Published:** 2015-03-07

**Authors:** Harriet E Downing, Fran Carroll, Sara T Brookes, Sandra Hollinghurst, David Timmins, Elizabeth Orton, Kay Wang, Denise Kendrick, Paul Little, Mike V Moore, Anthony Harnden, Matthew Thompson, Margaret T May, Alastair D Hay, on behalf of the OSAC trial team

**Affiliations:** Centre for Academic Primary Care, School of Social and Community Medicine, University of Bristol, Canynge Hall, 39 Whatley Road, Clifton, Bristol BS8 2PS UK; Nuffield Department of Primary Care Health Sciences, New Radcliffe House, Radcliffe Observatory Quarter, Woodstock Road, Oxford, OX2 6GG UK; Division of Primary Care, University of Nottingham, Floor 13, Tower Building, University Park, Nottingham, NG7 2RD UK; University of Southampton, Aldermoor Health Centre, Aldermoor Close, Southampton, SO15 5ST UK

**Keywords:** Acute cough, Lower respiratory tract infection, Oral steroids, Corticosteroids, Prednisolone, Randomised controlled trial, Efficacy, Cost-effectiveness

## Abstract

**Background:**

Acute lower respiratory tract infection (LRTI) is one of the most common conditions managed internationally and is costly to health services and patients. Despite good evidence that antibiotics are not effective for improving the symptoms of uncomplicated LRTI, they are widely prescribed, contributing to antimicrobial resistance. Many of the symptoms observed in LRTI are mediated by inflammatory processes also observed in exacerbations of asthma, for which there is strong evidence of corticosteroid effectiveness. The primary aim of the OSAC (Oral Steroids for Acute Cough) Trial is to determine whether oral prednisolone (40 mg daily for 5 days) can reduce the duration of moderately bad (or worse) cough and the severity of all its associated symptoms on days 2 to 4 post-randomisation (day 1 is trial entry) by at least 20% in adults ≥18 years with acute LRTI presenting to primary care.

**Methods/design:**

OSAC is a two-arm, multi-centre, placebo-controlled, randomised superiority trial. The target sample size is 436 patients, which allows for a 20% dropout rate. Patients will be recruited from primary care sites (General Practitioner surgeries) across England and followed up until symptom resolution. The two primary clinical outcomes are the duration of moderately bad (or worse) cough, and the severity of all its associated symptoms on days 2 to 4 post-randomisation. Secondary outcomes include: antibiotic consumption; symptom burden; adverse events; participant satisfaction with treatment and intention to consult for future similar illnesses. A parallel economic evaluation will investigate the cost-effectiveness of the intervention.

**Discussion:**

Results from the OSAC trial will increase knowledge regarding the clinical and cost-effectiveness of corticosteroids for LRTI, and will establish the potential of a new treatment option that could substantially improve patient health. We have chosen a relatively high ‘efficacy dose’ as this will enable us to decide on the potential for further research into lower dose oral and/or inhaled corticosteroids. This trial will also contribute to a growing body of research investigating the natural course of this very common illness, as well as the effects of steroids on the undesirable inflammatory symptoms associated with infection.

**Trial registration:**

Current Controlled Trials ISRCTN57309858 (31 January 2013).

**Electronic supplementary material:**

The online version of this article (doi:10.1186/s13063-015-0569-5) contains supplementary material, which is available to authorized users.

## Background

### Epidemiology, costs and current management of lower respiratory tract infection

Lower respiratory tract infection (LRTI), defined as an acute cough with at least one of sputum, chest pain, shortness of breath and/or wheeze [1], is one of the most common conditions managed in primary care in the UK and internationally [2]. Over 75% of patients presenting to primary care for this condition are prescribed antibiotics [3,4] and have a 20% likelihood of re-consulting within the same illness episode [1]. Acute cough is estimated to cost the UK National Health Service (NHS) at least £20 million annually in prescription costs and £170 million in consultation costs [5,6]. Patient costs have been shown to be in excess of £15 per episode [7], and respiratory tract infection is one of the most common reasons for work absenteeism [8].

Despite good evidence that antibiotics do not reduce the duration or severity of LRTI symptoms [2,5,9], they continue to be widely prescribed [2,5]. This is adding to the significant rise in antimicrobial resistance [10], which is an increasing and serious threat to public health [11,12]. Novel therapeutic measures are urgently needed [6], and so far evidence of efficacy for alternative treatments has not been demonstrated [13].

### Rationale for testing the effectiveness of corticosteroids in lower respiratory tract infection

Symptoms of LRTI include cough, wheeze and shortness of breath, which are similar to the symptoms of exacerbated asthma [14,15]. Evidence suggests that the worst symptoms may last up to 1 week, while complete symptom resolution may take 3 weeks or more [5]. Prolonged symptoms are thought to be due to a transient bronchial hyper-responsiveness [16,17] and experimental evidence suggests similar changes to bronchial epithelium in people with and without asthma during a respiratory tract infection. Both groups have been shown to have significant reductions in Forced Expiratory Volume (FEV), as well as airways inflammation [14].

Oral and inhaled corticosteroids are widely prescribed for the treatment of acute and chronic asthma, respectively, and work by exerting an array of anti-inflammatory effects [18]. There is increasing interest in the potential role of corticosteroids in modifying the undesirable effects of infection-associated inflammation [19]. Benefits have already been demonstrated for children with acute croup [20] and community-acquired pneumonia (if also given β agonists) [21], adults hospitalised with community-acquired pneumonia [22], adults with post-infectious persistent cough [23] and adults with acute tonsillitis [24].

At the time of writing there have been no published studies of oral corticosteroid therapy for acute cough following respiratory tract infection [6]; however, a number of trials of inhaled corticosteroids have been conducted, of which one [25] is relevant to the Oral Steroids for Acute Cough (OSAC) trial. This tested the effects of high-dose fluticasone, 1 mg twice daily, (equivalent to 2 mg twice daily of beclometasone [26] or 8.5 mg of oral prednisolone [27]) for 2 weeks in non-asthmatic adults presenting to Dutch primary care with a cough (LRTI) lasting more than 2 weeks. The inhaled corticosteroids were effective in reducing the mean cough frequency score among non-smokers, but the clinical importance of the reduced cough score is uncertain and there was no economic evaluation.

We are aware of two other ongoing trials currently registered on the International Controlled Trials Register [28] to investigate the value of corticosteroids for other infectious illnesses in primary care: the TOAST (Treatment Options without Antibiotics for Sore Throat) trial (ISRCTN17435450); and the OSTRICH (Oral STeroids for Resolution of otitis media with effusion In CHildren) trial (ISRCTN49798431).

Research and anecdotal evidence suggests that European clinicians have started prescribing corticosteroids for LRTI (in the absence of chronic obstructive pulmonary disease (COPD)) [29], even though there is limited evidence to support their use for this condition.

Long-term steroid use is known to be associated with an array of unwanted systemic side effects such as adrenal suppression, impaired skin collagen synthesis and metabolic disturbances [30,31]. However, in the absence of specific contraindications [30,32], a short (up to 1 week) course of high-dose corticosteroids is considered to be safe and associated with few side effects [32-34].

The rationale for testing the effectiveness of corticosteroids in LRTI can be summarised as follows: (i) there is good evidence of oral steroid effectiveness for acute asthma; (ii) the symptoms of LRTI overlap with those of acute asthma; (iii) prednisolone (tablets at a dose of 40 mg daily for 5 to 7 days) is the most commonly used oral steroid for acute asthma; (iv) there is pharmacokinetic evidence to suggest that a minimum dose of 20 mg daily is required for non-asthmatic patients [35]; and (v) it is important that the first trial of its kind uses an adequate dose to detect any potential effects as a proof of concept.

### Rationale for the trial design

This double-blind randomised controlled trial will provide high quality evidence to determine whether steroids are effective in the symptomatic treatment of acute LRTI, for which, to date, non-steroidal anti-inflammatory drugs [13], antibiotics [9] and inhaled corticosteroids [6] have been shown to be ineffective. A double-blinded, maximum dose design has been chosen since: (i) the primary outcomes are subjective (in that cough severity is reported by the participant); and (ii) treatment with this agent for this clinical problem is novel, making a trial demonstrating effectiveness under optimal conditions important.

‘Duration of moderately bad or worse cough’ and ‘mean severity of all LRTI symptoms on days 2 to 4 post-randomisation’ have been selected as the primary outcomes for this trial, in order to test the hypothesis that the anti-inflammatory effects of corticosteroids will relieve both the duration and severity of the unwanted bronchospasm and other inflammatory side-effects of infection. In another study, participants reported symptoms between days 2 and 4 as being the worst problem [2], which provides the rationale for the timing for the second primary outcome in this trial.

We recognise the undesirability of further medicalisation of common and self-limiting infections in primary care [36], and if this trial demonstrates a clinically important treatment effect, we do not think it will be appropriate to promote the routine use of high-dose corticosteroids for acute LRTI (though we recognise that the prescription of corticosteroids to alleviate the most acute symptoms of chest infections remains a clinical decision). Rather, we think the implication will be that further trials of lower-dose oral or inhaled corticosteroids should be conducted. If no treatment effect is found, it is unlikely that further, lower-dose steroid research would be warranted for acute LRTI.

### Research questions

#### Primary research question

Can the use of oral prednisolone reduce the duration of moderately bad or worse cough and/or the severity of all its associated symptoms on days 2 to 4 post-randomisation by at least 20% when compared to placebo treatment in adults 18 years and over presenting to primary care with acute LRTI?

#### Secondary research questions

We will also: 1) assess the effects on antibiotic consumption; 2) estimate the cost-effectiveness from the perspectives of the NHS, patients, and society; 3) compare the burden, severity and duration of abnormal peak flow and LRTI symptoms; 4) compare adverse events including any new symptoms or worsening of existing symptoms, re-consultations for a documented deterioration in illness and serious adverse events; 5) investigate if participants’ subjective or objective response to oral steroids is associated with a clinical diagnosis of asthma or COPD; and 6) assess participants’ satisfaction with treatment and intention to consult for future similar illnesses.

## Methods/design

This study is a placebo-controlled, individually randomised, superiority trial in UK general practice.

### Eligibility

We wish to test the effects of corticosteroids in adults presenting to primary care with acute LRTI, in whom there is no evidence of pneumonia or other reason to require an immediate antibiotic or hospitalisation, and in whom there is no reason to consider the use of oral prednisolone 40 mg daily for 5 days unsafe. At the same time, the relatively high dose used in this trial requires stringent exclusion criteria to ensure patient safety. The eligibility criteria are as follows:

#### Inclusion criteria

The following inclusion criteria must all apply:

1. Aged 18 years or over;
2. Consulting for an acute (≤28 days) cough as the main presenting symptom;
3. In the past 24 hours, the patient has had at least one of the screening symptoms listed below (a-d), localising to the lower respiratory tract and suggestive of an acute LRTI:
  - phlegm (sputum)
  - chest pain
  - shortness of breath
  - wheeze
4. Patient and practice have sufficient time for consent and randomisation into the trial by the end of the day of consultation, or the next working day as long as this is within 24 hours;
5. Patient able and willing to give informed consent themselves;
6. Patient able and willing to complete the daily symptom diary themselves;
7. Patient able, willing and available to receive weekly telephone calls from the trial team.

#### Exclusion criteria

The presence of any of the following exclusion criteria warrants exclusion:

1. Known lung cancer or chronic lung disease (for example, cystic fibrosis, COPD, bronchiectasis);
2. Has an ‘active’ diagnosis of asthma (for which any treatment has been given in the past 5 years);
3. The patient’s LRTI warrants same day hospital admission or immediate antibiotics (note: use of delayed prescription (post-dated by at least one working day after the randomisation date) does not preclude OSAC trial participation):
  According to NICE guidelines, the patient warrants immediate antibiotic treatment by virtue of one or more of the following:
  A. Is clinically very unwell or has symptoms and signs suggestive of pneumonia, for example tachypnoea (>20 bpm), unilateral chest signs or consolidation, or hypoxia (oxygen saturation <94%) or other systemic infection, for example suspected bacteraemia
    or
  B. Is at high risk of complications, including patients with chronic heart, chronic lung (for example, COPD, bronchiectasis and cystic fibrosis), chronic renal, chronic liver or neuromuscular disease or immunosuppression; or with complications from previous episodes of lower respiratory tract infection, for example hospital admission for pneumonia
    or
  C. Aged over 65 years with at least two of the following criteria, or aged over 80 years with at least one of the following criteria:
    I. Unplanned hospitalisation within the previous year
    II. Type 1 or Type 2 diabetes
    III. History of cardiac failure
4. Requires an oral or systemic antibiotic on the day of consultation to treat another infection unrelated to their acute cough, for example a co-existing cellulitis (note: use of topical antibiotics does not preclude OSAC trial participation);
5. Recently (≤1 month) used inhaled corticosteroids;
6. Recently (≤1 month) used short- (up to 2 weeks) course systemic corticosteroids;
7. Currently using, or has previously (≤12 months) used systemic steroids for a cumulative period greater than 2 weeks, that is “long-term” use;
8. Known to be pregnant, is trying to conceive or is at risk of pregnancy (for example, unwilling to take a reliable form of contraception) in the next month;
9. Currently breast-feeding;
10. This is not the patient’s usual practice, that is the patient is visiting or is not intending to stay with the practice for the 3 month trial follow-up period;
11. Previously randomised into the OSAC trial;
12. Has been involved in another medicinal trial within the last 90 days or any other clinical research study within the last 30 days;
13. Is unable to give informed consent or complete the trial paperwork (including the symptom diary) through mental incapacity, for example major current psychiatric illness, learning difficulties and dementia;
14. Known immune-deficiency, for example chemotherapy causing immunosuppression, asplenia or splenic dysfunction, advanced cancer or HIV infection;
15. Patient due to receive the shingles vaccine in conjunction with the influenza vaccine;
16. Has any of the following (A-P) known contraindications or cautions to oral steroids:
  Current or previous history of:
    A. Peptic ulcer disease
    B. Previous tuberculosis (TB)
    C. No previous chickenpox and known recent (≤28 days) history of close personal contact with chickenpox or herpes zoster
    D. Known allergy to prednisolone or other OSAC trial tablet ingredients (potato starch, lactose monohydrate, colloidal silicon dioxide, sodium starch glycolate, magnesium stearate), galactose intolerance, Lapp lactase deficiency or glucose-galactose malabsorption
    E. Osteoporosis
    F. Glaucoma
    G. Suspected ocular herpes simplex
    H. Cushing’s disease
    I. Epilepsy
    J. Severe affective disorders, for example manic depression, previous steroid psychosis
    K. Previous steroid myopathy
    L. Intention to use a live vaccine in the next 8 weeks or has received a live vaccine in the previous 2 weeks (note: assess live vaccine status by cross-checking with the British National Formulary)
  Current history only:
    M. Uncontrolled diabetes (glycated haemoglobin or HbA1C >8%)
    N. Uncontrolled hypertension (note: as per Responsible Clinician’s routine clinical judgement)
    O. Taking other interacting medication (e.g. phenytoin and anti-coagulants)
    P. Any other British National Formulary listed contraindication or caution (note: as per Responsible Clinician’s routine clinical judgement)
17. Is unable to swallow tablets

To meet the recruitment target and to ensure a generalisable patient population, recruitment will take place across four collaborating UK trial centres: the Universities of Bristol, Nottingham, Oxford and Southampton.

### Intervention and blinding

Participants will be randomly assigned to one of two treatments: (i) 2 × 20 mg oral prednisolone tablets daily for 5 days or (ii) 2 × 20 mg oral placebo tablets daily for 5 days. Participants may stop taking the trial medication before the 5 days if they feel completely better for two consecutive days. Participants, clinicians and the trial team (including the statisticians) will all be blinded to allocation. All participants randomised to the trial will continue to receive usual clinical care.

### Treatment allocation, concealment and emergency unblinding

The computer-generated randomisation schedule will be produced by a statistician who is independent of the OSAC trial statisticians, and stratified by centre using a variable block size. The schedule will be held by the Pharmacy of the University Hospitals Bristol NHS Foundation Trust (“the Pharmacy”), who will allocate medicine packs containing active and placebo tablets and identified by a unique Medicine ID number, to identical, sequentially numbered Patient Packs identified by a unique Participant ID number, which will then be sealed. Packs, which are indistinguishable between active and placebo groups, are issued sequentially to eligible, consented patients at recruiting primary care sites. Clinicians, patients and all members of the research team were masked to the randomisation sequence, and all outcome data were gathered masked to allocation status.

The use of distinct Participant ID and Medicine ID numbers will enable flexibility in the number of patients recruited at each of the four trial centres. Medicine packs can be combined with patient packs as needed, allowing for temporary differential fluctuations in recruitment rates between centres. The four centres will be provided with Patient Packs to distribute to the participating primary care sites in blocks of four (although larger numbers of packs will be issued to practices with proven capacity to recruit to this trial).

The Pharmacy will hold the master drug allocation log and provide a 24 hour emergency unblinding service based on a standard operating procedure for breaking the code in the event of a medical emergency. Trial participants will be given a Trial Participation Card with details of who their Responsible Clinician should contact in the event of an emergency, and all practice-based clinicians will receive training in the use of the emergency unblinding service.

At the end of the trial, the code-break will only be released to the investigative team once written confirmation has been received that primary outcome data analysis is complete.

### Outcomes

#### How the outcome measures will be ascertained

The OSAC trial will use validated patient completed symptom diary methods [37] that have been used in a number of similar previous trials [2,5]. Participants will record in the diary the severity of the following symptoms: cough; phlegm; shortness of breath; sleep disturbance; feeling generally unwell; activity disturbance. A symptom score scale of 0 to 6 will be used (0 = no problem, 1 = very little problem, 2 = slight problem, 3 = moderately bad, 4 = bad, 5 = very bad, and 6 = as bad as it could be) which is shown to be sensitive to change and internally reliable [5]. For this trial, we will record all the above symptoms for up to 28 days (or until symptom resolution for two consecutive days) since LRTI duration has been previously shown to last 3 to 4 weeks [5]. Cough duration and severity will be measured for up to 8 weeks since effects on these may not be apparent for some time after using corticosteroids.

For this trial, participants will also record their peak expiratory flow in the morning and the evening for the duration of their illness up to 28 days, and measure their quality of life at weekly intervals (up to 4 weeks) using the EQ-5D-5L validated questionnaire [38], which has been shown to be moderately responsive in participants with acute cough/LRTI, and is a suitable measure for use in economic evaluation studies of this illness [39].

#### Primary outcome measures

1. Duration of moderately bad or worse cough (using a validated web/paper-based symptom diary containing an item asking participants to rate their cough from 0 to 6 (as described in ‘How the outcome measures will be ascertained’ above). Duration is calculated as the number of days from randomisation to the last day that the participant scored 3 or higher, where that last day is followed by at least two consecutive days where the score is less than 3;
2. Mean of all symptom severity scores on days 2 to 4 post randomisation, measured using the symptom diary. Severity of symptoms is scored 0 to 6 as for cough, detailed above. Symptoms include cough, phlegm, shortness of breath, disturbed sleep, feeling generally unwell, and interference with normal activities/work. A mean score will be calculated across these symptoms for each of days 2, 3 and 4 and then an overall mean calculated.

#### Secondary outcome measures

1. Antibiotic consumption;
2. Duration of corticosteroid tablet use;
3. Total duration and severity of symptoms (cough; phlegm; shortness of breath; wheeze; blocked/runny nose; chest pain; fever; muscle aching; headache; sleep disturbance; feeling generally unwell; activity disturbance), until the severity of each is scored by the participant as ‘1’ or ‘very little problem, and abnormal peak flow (defined as a peak flow reading that is not within the range of values that would be expected according to a standard predictive algorithm based on gender, height and weight);
4. Adverse events including re-consultation for a documented illness deterioration;
5. Patient satisfaction with treatment and intention to use the same treatment if it were to be available in the future;
6. Clinical diagnosis of asthma, COPD, whooping cough (pertussis), or lung cancer at 3 months;
7. Quality of life (using the EQ-5D-5L);
8. NHS treatment and investigation costs, out-of-pocket patient and family costs, and the cost to society of time off work.

### Sample size calculation

Since the distributions of both primary outcome variables (the duration of moderately bad or worse cough and the mean severity score of all its associated symptoms on days 2 to 4 post-randomisation) will be positively skewed, sample size calculations are based on the log-normal distribution.

The mean (standard deviation (SD)) duration of bad or moderately worse cough was taken from a previous study examining the effectiveness of prescribed antibiotics for acute LRTI [5] and estimated as 5.8 (4.1) days. The mean (SD) symptom severity score between days 2 and 4 taken from the same trial was estimated as 2.3 (1.1). Using standard formulae for the mean and standard deviation of a log-normal distribution, this corresponds to 1.555 (SD 0.637) log days (or geometric mean of 4.74 days) for the cough duration outcome and 0.73 (SD 0.454) on the log scale (or geometric mean of 2.08) for the symptom severity outcome.

To account for testing multiple primary outcomes a lower alpha was specified for the symptom severity outcome to ensure that the overall alpha for the two primary outcomes remains close to 5%. Moderately bad or worse cough has been shown to resolve within 7 days for 50% of patients, 14 days for 75% and 4 weeks for 90% of patients [5]. Therefore, since duration of cough lasts significantly longer than the period during which severity of symptoms are measured (days 2 to 4) and is capturing an element of the illness severity, duration of moderately bad or worse cough might be viewed as the slightly more clinically important outcome. For this reason alpha remained at 5% for duration of cough and was reduced to 0.1% for symptom severity.

Sample size was calculated based on a 20% reduction in the duration of cough, corresponding to a geometric mean in the active treatment group of 3.79 days (mean 1.333 log days). Allowing (conservatively) for 20% attrition, 218 participants will need to be randomised per arm to retain 174 at follow-up and achieve 90% power with a two-sided alpha of 0.05. Hence a total sample size of 436 is required.

For the symptom severity outcome, a final achieved sample size of 174 participants in each arm will provide 89% power to detect a reduction in symptom severity score between days 2 and 4 of 20%, corresponding to a geometric mean of 1.66 units in the active group (mean 0.51 log units) with a two-sided alpha of 0.001.

The trial team will make all efforts to recruit the 436 participants. If recruitment is slower than anticipated, recruitment will be extended until at least 163 have been recruited to each arm (326 in total). Assuming 20% attrition, this would provide the study with 80% power for both primary outcomes. All attempts will be made to ensure that attrition remains below 20% but if this is not feasible recruitment will be extended further if possible.

### Trial medicines

The Investigational Medicinal Product for this trial will be Prednisolone 20 mg oral tablets (×10), procured from GALEN Pharma GmbH (Kiel, Germany). Placebo tablets will be manufactured by Piramal Healthcare Ltd (Morpeth UK) to exactly match the prednisolone tablets in dimensions, appearance and taste, to maintain allocation blinding.

A relatively high dose of corticosteroids has been selected in order to maximise the probability of detecting a treatment effect (that is, an ‘efficacy’ dose) such that a negative result cannot be criticised for being due to an inadequate dose. The patient’s weight and height will be recorded in order to allow us to take account of dose effects.

### Recruitment sites

A minimum of 60 GP practices will be recruited to take part in the trial across the four trial centres, with a wide geographical spread in both urban and rural areas across the South West, Midlands and North West of England. The number recruited will take account of anticipated ready to recruit (open) to actually recruiting (active) ratios. Participating practices are required to have recent (within 5 years) Good Clinical Practice (GCP) training for all practice staff who will be confirming patients’ eligibility, authorising the trial prescription, consenting patients or entering clinical data onto the online database.

### Research ethics and governance

Multi-centre approval was granted by the Central Bristol Research Ethics Committee (Ref: 12/SW/0180) and by the MHRA (EudraCT number: 2012-000851-1). The lead Research and Development organisation was the Western (now West of England) Clinical Research Network. The following Clinical Commissioning Groups provided Research and Development approval for OSAC trial recruitment at primary care sites within their localities: Bristol (lead Clinical Commissioning Group); Berkshire East; Birmingham CrossCity; Blackburn with Darwen; Bracknell and Ascot; Coventry and Rugby; Cumbria; Dorset; East Lancashire; Fylde & Wyre; Gloucestershire; Hardwick; Kernow; Lancashire North; Leicester City; Nene; North and West Reading; North Derbyshire; North East Hampshire and Farnham; North Hampshire; North Somerset; Northern, Eastern and Western Devon; Nottingham City; Oxfordshire; Portsmouth; Redditch and Bromsgrove; Sandwell and West Birmingham; Somerset; South Devon and Torbay; South Eastern Hampshire; South Warwickshire; Warwickshire North; West Hampshire; Wiltshire; Wokingham.

Please see Additional file 1 (SPIRIT checklist) for a summary of how the protocol meets the recommendations of the SPIRIT (Standard Protocol Items: Recommendations for Interventional Trials) initiative.

### Screening for eligibility (the routine consultation)

Patients presenting with cough will be approached by the healthcare professional managing their clinical care (‘Responsible Clinician’) or by another member of the practice team responsible for patient identification (for example, reception staff or the ‘Recruiting Clinician’), given a short explanation of the trial, and invited to be screened for eligibility. If the patient is willing, the clinician will screen the patient for eligibility. This process includes a detailed check of the inclusion/exclusion criteria, and a routine clinical examination, of which the results and the clinician’s diagnosis will be recorded on the case report form (CRF). The routine clinical management of the patient will be completed as normal. This may include giving the patient a discretionary delayed antibiotic prescription (to be used if the patient’s condition deteriorates after 48 hours or if failing to improve after 7 days) and the discretionary use of a β-agonist (for example, salbutamol). A requirement for immediate antibiotic treatment renders the patient ineligible.

Once the patient’s eligibility is confirmed, the trial prescription will be authorised (by a GCP-trained GP) for redemption (by the Recruiting Clinician, not the patient) against a trial Patient Pack. The patient is then referred to a further interview for full recruitment and trial entry.

### Trial entry (the recruitment interview)

The recruitment interview must take place on the same, or next, day as the routine consultation. Same-day recruitment will be more efficient for many patients who may not wish to return to the GP practice again the following day. Same-day recruitment will also help ensure that participants take the first dose of their trial medication prior to collecting any delayed antibiotic that may have been prescribed for them by their GP, thereby ensuring the validity of antibiotic consumption as one of the secondary outcome measures. Some patients may wish to start taking a treatment on the same day that they visit the GP practice, and in the absence of a trial treatment those patients may be more likely to take antibiotics obtained from a delayed prescription or visited another healthcare provider while waiting for the next day to enter the trial.

However, if the site or patient do not have time for the recruitment interview on the same day, the patient can be recruited on the following working day, if the following conditions are met: the recruitment interview is not deferred to the Monday following a weekend, to reduce the possibility of recruiting patients whose clinical condition has deteriorated significantly since the eligibility assessment was performed; and any delayed antibiotic prescription is post-dated by at least 24 hours after the recruitment interview, to ensure that the patient has the opportunity to take the first dose of their trial medication prior to collecting their delayed antibiotic prescription.

The Recruiting Clinician will take formal written consent, collect the remaining CRF data (including symptoms, signs and respiratory history), issue the patient with the trial medicines and explain how to take them. The patient will also receive a symptom diary, peak flow meter and other materials to fully equip them for the follow-up, and training in how to measure peak expiratory flow and complete the diary.

### Participant follow-up

All follow-up will be managed by the trial team, who will give participants individual support throughout their follow-up period. Participants will complete the symptom diary online or on paper every day for up to 28 days or until symptoms have been completely resolved for two consecutive days (whichever is soonest). As well as recording the severity of their symptoms, completing the symptom diary includes recording peak flow measurements and whether or not any antibiotics have been taken. The patient will also record how many of the trial tablets they took on days 1 to 5, and any adverse effects during the first 7 days.

All participants will be telephoned within the first 2 days of trial entry, and then each week for 4 weeks to support symptom diary completion, collect the daily data they have recorded during the preceding week (to safeguard against potential loss of data if paper diaries are not returned to the trial centre or there are problems with the post), and to collect the weekly data on resource use and quality of life measures. These methods are similar to those successfully used (with <20% attrition) in previous studies [2,5,40,41].

Should the patient’s cough persist beyond 28 days, their permission will be sought to continue with the weekly calls, up to a maximum of 56 days, in order to establish the day on which the patient last scores their cough as “moderately bad”, and the date on which their cough is completely resolved. The 56 day cut-off point has been chosen as a pragmatic cut-off point, in order to establish the date of cough resolution for the majority of participants while taking account of the participant’s research burden, and of the trial resources. The patient will not be asked to complete any further trial paperwork after the initial 28 day period.

Participants will receive thank you vouchers worth £15 during their participation in the trial, as there is systematic review evidence that small monetary tokens increase response rates [42].

A review of participants’ primary care notes will be undertaken by the recruiting primary care site, to record NHS contacts (and their causes), prescriptions, secondary care referrals and any clinical diagnoses of asthma, COPD, whooping cough or lung cancer in the 3 months post-randomisation. This data collection will take place at least 4 months after randomisation to allow for secondary care contacts and test results to be evident in the primary care notes.

**Figure 1:**
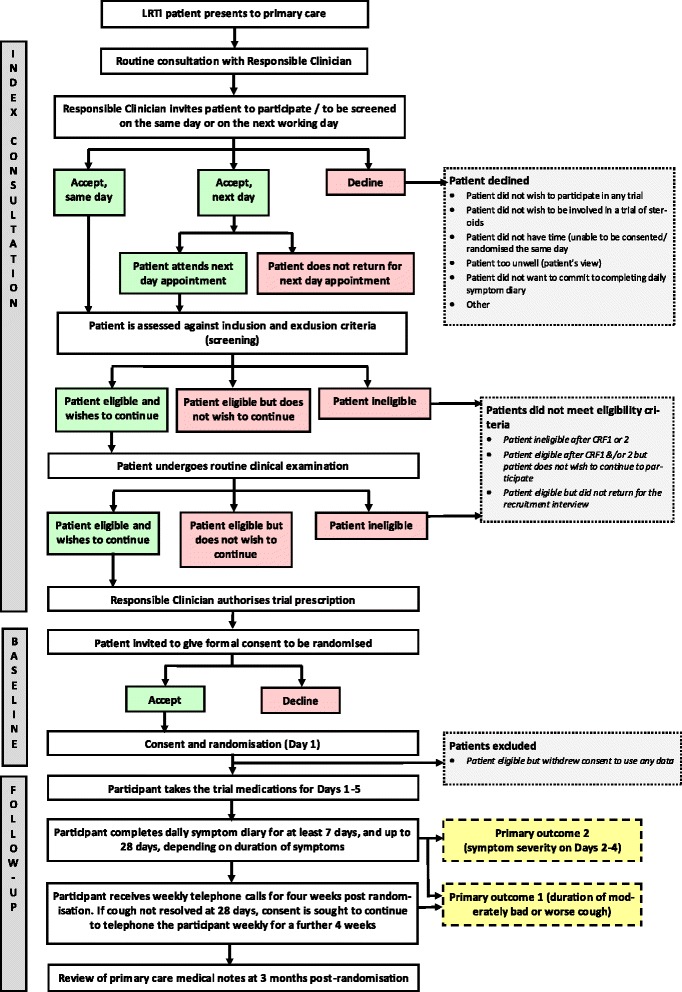
**Study flow chart.** A visual representation of the pathway of the trial participant through the trial (presentation, index consultation, baseline recruitment interview, and follow-up). CRF, case report form; LRTI, lower respiratory tract infection.

See Figure 1 (study flow chart) for a visual representation of the pathway of the trial participant through the trial (presentation, index consultation, baseline recruitment interview, and follow-up).

### Data management

Clinical data will be collected and managed using a secure, web-based system (OpenClinica) which will be developed, hosted and supported by the University of Oxford and validated by the University of Bristol. Patient confidentiality will be maintained at all stages of data collection. Eligible, consented, randomised patients will be identified by the Participant ID number and the Medicine ID number of the allocated Patient Pack. Entry of data into the on-line database will be the default method of data capture; however, paper-based alternatives will be provided in order to accommodate GPs’ individual preferences. Participants will also be given the choice between online and paper data collection: the symptom diary will be made available online, with the exception of the EQ-5D-5L which will be completed by the patient on paper and also collected by the Trial Research Nurse during the weekly telephone calls.

### Analysis

Descriptive analyses will examine the comparability of the intervention and control group at baseline.

#### Analysis of primary outcomes

Analysis and reporting will be in line with CONSORT guidelines [43]. The primary comparative analysis will be conducted on an intention-to-treat (ITT) basis. For all analyses effect estimates will be presented with 95% confidence intervals and *P* values. For the primary analysis missing data will be assumed to be missing at random and excluded from the analysis.

Duration of cough is calculated as the number of days from the index consultation to the last day that the patient scored 3 or higher, where that last day is followed by at least two consecutive days with a score of less than 3. Cox proportional hazards models (adjusting by centre) will be employed to examine differences in time to recovery from moderately bad to worse cough between the two groups. Individuals not recovered at 28 days post-randomisation will be censored at this time point. The assumption of proportional hazards will be checked using Schoenfeld residuals.

Multiple linear regression models (adjusting for centre) will evaluate the effectiveness of steroids in terms of reducing symptom severity (the mean score of six symptoms) on days 2 to 4.

For both primary outcomes models will also adjust for any covariates demonstrating imbalance between the groups at baseline.

Factors associated with missing data (such as demographics and values of primary and important secondary outcome variables at baseline) will be explored and sensitivity analyses conducted (including inverse probability weights or multiple imputation methods, depending on whether outcome data is partially or fully missing).

It is anticipated that not all participants will complete the full course of tablets; hence, in further sensitivity analysis a per-protocol analysis will be performed.

#### Analysis of secondary outcomes

The secondary outcomes listed above (see “Secondary outcome measures”) will be analysed using appropriate regression models and will adjust for the baseline measure of the outcome, where possible, and centre.

#### Subgroup analyses

The following potential effect modifiers will be examined by formal tests of interaction: age, duration of illness (both using the median from the completed dataset), presence of wheeze on auscultation, antibiotic consumption, β-agonist prescriptions, smoking status, history of hay fever, allergic rhinitis, eczema, and diagnosis of asthma or COPD at 3 months (all yes/no). The trial is not specifically powered for these analyses; interaction tests will therefore be performed as hypothesis-generating analyses and interpretation will focus on 95% confidence intervals.

#### Economic analysis

The economic analysis will use patient level data on participant resource use over the 28 day period between randomisation and the final follow-up telephone call. This will be compared with outcomes measured at the 28-day follow-ups. The analysis will consider three perspectives: (1) the health care provider and personal and social services (NHS and PSS); (2) participants and their families, (3) societal cost of lost productivity due to time off work.

The costs associated with the NHS and PSS perspective will include: trial and prescription medication costs, and the costs associated with primary and secondary care consultations. Participant resource use will include travel to consultations, expenditure on over-the-counter medications, cost of extra domestic help and childcare, prescription payments, and loss of earnings. The impact on society due to time off work will be captured by participants’ reports of their own and their families’ absenteeism.

We will use the data listed above to construct a cost-consequences matrix comparing cost from the three perspectives with the full range of primary and secondary outcomes. We will estimate incremental cost-effectiveness ratios comparing the extra cost, from the NHS perspective, of treating participants in the intervention group, with the extra benefit gained. Benefit will be described as (i) reduced cough duration, defined as the percentage of participants in each group with a duration at least 20% lower than the mean duration of participants in the control group or (if 20% reduction in mean duration in the control group is less than 1 day) the percentage of participants in each group whose duration is at least 1 day less than the mean of those in the control group; and (ii) quality-adjusted life year (QALY) gain (QALY estimates will be based on responses to the EQ-5D-5 L).

Sensitivity analyses will be used to test the robustness of the results against assumptions made and bootstrapping will be used to estimate the level of uncertainty around the estimates of cost per QALY.

## Discussion

This paper describes a placebo-controlled, randomised multi-centre superiority trial that will establish the clinical and cost effectiveness of a commonly used treatment (corticosteroids) for an entirely novel indication and one of the commonest clinical problems managed in primary care: acute LRTI. The trial will recruit between 326 and 436 non-asthmatic adult patients presenting to primary care with an acute cough of less than 28 days duration and at least one other lower respiratory tract symptom or physical examination finding. Eligible, consented patients will be randomised to receive a 5-day course of either the active treatment (2 × 20 mg prednisolone daily) or matched placebo, and asked to complete a symptom diary for at least 7 and up to 28 days, depending on the duration of their illness. Participants will be telephoned weekly for 4 weeks, or until their cough resolves, up to a maximum of 8 weeks from recruitment.

## Trial status

The OSAC trial completed recruitment on 27 October 2014.

## Additional file

Additional file 1:
**SPIRIT checklist.**

## Abbreviations

COPD: chronic obstructive pulmonary disease
CRF: case report form
GCP: Good Clinical Practice
GP: General Practitioner
LRTI: lower respiratory tract infection
NHS: National Health Service
OSAC: Oral Steroids for Acute Cough
QALY: quality-adjusted life year
SD: standard deviation
